# Supplementary material for: Fermentation characteristics of bedded pack barn dairy cattle manure on methane yield, carbon, and nitrogen content in solid-state anaerobic digestion
Source: PeerJ. 2022 Oct 14;10:e14134. doi: 10.7717/peerj.14134 (PMC9575680; doi:10.7717/peerj.14134)
Supplement: Supplemental Information 1 [file peerj-10-14134-s001.docx]

**APPENDIX**

| Table A.1.  Changes in the daily and cumulative methane production of bedded pack barn dairy cattle manure based on digestion time in the batch solid-state anaerobic digestion (n=3)  (Mean ± SD) | | | | | | |
| --- | --- | --- | --- | --- | --- | --- |
| Day | Daily CH4 production | | | Cumulative CH4 production | | |
|  | ------------------------------ N·mL / g VS ------------------------------ | | | | | |
| 0 | 0.0 | ± | 0.0 | 0.0 |  | 0.0 |
| 3 | 1.2 | ± | 0.5 | 1.3 | ± | 0.1 |
| 6 | 1.7 | ± | 1.0 | 2.9 | ± | 0.9 |
| 9 | 1.0 | ± | 0.5 | 4.7 | ± | 0.6 |
| 12 | 1.0 | ± | 0.4 | 5.7 | ± | 1.0 |
| 15 | 5.5 | ± | 4.2 | 14.6 | ± | 4.9 |
| 18 | 13.7 | ± | 7.2 | 31.2 | ± | 8.2 |
| 21 | 13.0 | ± | 6.7 | 49.4 | ± | 9.0 |
| 24 | 12.8 | ± | 7.0 | 66.5 | ± | 7.5 |
| 27 | 16.7 | ± | 6.7 | 86.5 | ± | 6.6 |
| 30 | 9.5 | ± | 5.4 | 98.6 | ± | 6.1 |
| 33 | 6.8 | ± | 2.7 | 106.1 | ± | 7.1 |
| 36 | 5.7 | ± | 2.4 | 112.9 | ± | 7.7 |
| 39 | 5.1 | ± | 1.0 | 118.4 | ± | 8.6 |
| 42 | 3.8 | ± | 1.9 | 123.1 | ± | 8.7 |
| 45 | 3.4 | ± | 1.7 | 127.4 | ± | 8.7 |
| 48 | 1.9 | ± | 1.2 | 129.9 | ± | 9.2 |
| 51 | 1.0 | ± | 0.5 | 131.4 | ± | 9.4 |
| 54 | 0.6 | ± | 0.5 | 132.4 | ± | 9.7 |
| 57 | 0.9 | ± | 0.2 | 133.4 | ± | 9.8 |
| 60 | 0.7 | ± | 0.3 | 134.3 | ± | 10.1 |
| 63 | 0.3 | ± | 0.0 | 140.6 | ± | 6.4 |
| 66 | 0.5 | ± | 0.3 | 141.5 | ± | 6.3 |
| 69 | 0.2 | ± | 0.0 | 141.8 | ± | 6.2 |
| 72 | 0.5 | ± | 0.0 | 142.5 | ± | 6.0 |
|  | | | | | | |
|  | | | | | | |
|  | | | | | | |

| Table A.2. Changes in the methane content of bedded pack barn dairy cattle manure based on digestion time in the batch solid-state anaerobic digestion (n=3)  (Mean ± SD) | | | |
| --- | --- | --- | --- |
| Day | Methane contents (%) | | |
| 0 | 0.0 | ± | 0.0 |
| 3 | 17.3 | ± | 6.4 |
| 6 | 32.2 | ± | 6.1 |
| 9 | 36.6 | ± | 7.5 |
| 12 | 41.4 | ± | 4.1 |
| 15 | 54.9 | ± | 3.7 |
| 18 | 63.1 | ± | 2.0 |
| 21 | 63.0 | ± | 1.5 |
| 24 | 62.2 | ± | 3.8 |
| 27 | 62.3 | ± | 2.5 |
| 30 | 63.7 | ± | 4.1 |
| 33 | 59.4 | ± | 3.9 |
| 36 | 59.3 | ± | 2.9 |
| 39 | 57.2 | ± | 1.4 |
| 42 | 57.3 | ± | 2.2 |
| 45 | 60.9 | ± | 2.5 |
| 48 | 61.5 | ± | 2.4 |
| 51 | 66.8 | ± | 7.1 |
| 54 | 63.3 | ± | 7.6 |
| 57 | 67.3 | ± | 3.6 |
| 60 | 66.8 | ± | 5.9 |
| 63 | 76.1 | ± | 7.3 |
| 66 | 66.3 | ± | 0.6 |
| 69 | 61.9 | ± | 13.5 |
| 72 | 66.7 | ± | 4.2 |
|  | | | |
|  | | | |

| Table A.3. Changes in the cellulose, hemicellulose, and lignin content of bedded pack barn dairy cattle manure based on digestion time in the batch solid-state anaerobic digestion^1^ (n=3)  (Mean ± SD) | | | | | | | | | |
| --- | --- | --- | --- | --- | --- | --- | --- | --- | --- |
| Day | Cellulose (% VS) | | | Hemicellulose (% VS) | | | Lignin (% VS) | | |
| 0 | 16.8 | ± | 0.5 | 15.4 | ± | 0.5 | 15.1 | ± | 1.0 |
| 3 | 16.5 | ± | 0.6 | 14.5 | ± | 0.3 | 13.8 | ± | 0.1 |
| 6 | 16.6 | ± | 0.5 | 13.9 | ± | 0.2 | 15.3 | ± | 0.5 |
| 12 | 16.5 | ± | 0.1 | 13.1 | ± | 0.1 | 13.7 | ± | 1.1 |
| 18 | 15.9 | ± | 1.3 | 13.2 | ± | 0.3 | 18.2 | ± | 6.2 |
| 24 | 14.9 | ± | 1.3 | 13.1 | ± | 0.4 | 24.6 | ± | 1.9 |
| 30 | 14.6 | ± | 0.2 | 13.2 | ± | 0.4 | 21.9 | ± | 4.2 |
| 36 | 15.1 | ± | 0.7 | 11.8 | ± | 0.9 | 16.4 | ± | 0.3 |
| 48 | 14.4 | ± | 0.3 | 9.9 | ± | 0.8 | 16.7 | ± | 0.2 |
| 60 | 13.7 | ± | 0.8 | 8.9 | ± | 0.5 | 19.8 | ± | 3.3 |
| 72 | 14.9 | ± | 0.7 | 8.9 | ± | 0.5 | 18.4 | ± | 0.2 |
| P-value |  |  |  |  |  |  |  |  |  |
| Linear | 0.001 | | | <0.001 | | | 0.001 | | |
| Quadratic | 0.009 | | | 0.622 | | | 0.005 | | |
| Cubic | 0.282 | | | 0.304 | | | 0.139 | | |
| ^1^% VS, Volatile solid basis. | | | | | | | | | |
|  | | | | | | | | | |

| Table A.4. Changes in the carbon content of bedded pack barn dairy cattle manure based on digestion time in the batch solid-state anaerobic digestion^1^ (n=3) | | | | | | | | | |
| --- | --- | --- | --- | --- | --- | --- | --- | --- | --- |
| Day | NFCF (% v.b.) | | | HCF (%v.b.) | | | ADIC (%v.b.) | | |
| 0 | 15.43 | ± | 0.43 | 18.83 | ± | 0.90 | 13.53 | ± | 0.07 |
| 3 | 14.00 | ± | 0.77 | 14.36 | ± | 0.60 | 8.94 | ± | 1.44 |
| 6 | 14.61 | ± | 0.74 | 12.20 | ± | 0.77 | 8.82 | ± | 0.21 |
| 12 | 15.09 | ± | 0.59 | 13.76 | ± | 0.49 | 9.87 | ± | 0.52 |
| 18 | 15.14 | ± | 1.44 | 14.06 | ± | 1.18 | 9.67 | ± | 1.10 |
| 24 | 12.47 | ± | 1.55 | 11.72 | ± | 0.34 | 10.14 | ± | 0.62 |
| 30 | 11.93 | ± | 0.78 | 11.26 | ± | 1.95 | 10.76 | ± | 1.15 |
| 36 | 12.53 | ± | 0.92 | 10.61 | ± | 1.14 | 9.05 | ± | 0.31 |
| 48 | 13.15 | ± | 0.71 | 10.31 | ± | 0.66 | 8.21 | ± | 0.47 |
| 60 | 12.57 | ± | 0.83 | 10.04 | ± | 0.64 | 7.19 | ± | 0.48 |
| 72 | 12.88 | ± | 0.33 | 9.80 | ± | 0.32 | 7.28 | ± | 0.20 |
| P-value |  |  |  |  |  |  |  |  |  |
| Linear | <0.001 | | | <0.001 | | | <.0001 | | |
| Quadratic | <0.001 | | | 0.003 | | | 0.614 | | |
| Cubic | 0.072 | | | 0.064 | | | 0.265 | | |
| NFCF, non-fibrous carbon fraction; HCF, hemicellulose carbon fraction; ADIC, Acid detergent insoluble carbon; % v.b., Volatile solid basis. | | | | | | | | | |
| ^1^NFCF = total carbon – HCF, HCF = neutral detergent insoluble carbon – ADIC, and ADIC included carbon content of cellulose and lignin content. | | | | | | | | | |

| Table A.5. Changes in nitrogen content of bedded pack barn dairy cattle manure based on digestion time in the batch solids-state anaerobic digestion^1^ (n=3) | | | | | | | | | |
| --- | --- | --- | --- | --- | --- | --- | --- | --- | --- |
| Day | NFNF (% v.b.) | | | HNF (% v.b.) | | | ADIN (% v.b.) | | |
| 0 | 1.59 | ± | 0.31 | 0.40 | ± | 0.00 | 0.13 | ± | 0.00 |
| 3 | 1.46 | ± | 0.21 | 0.25 | ± | 0.06 | 0.25 | ± | 0.06 |
| 6 | 1.37 | ± | 0.11 | 0.21 | ± | 0.12 | 0.21 | ± | 0.03 |
| 12 | 1.26 | ± | 0.07 | 0.23 | ± | 0.14 | 0.23 | ± | 0.10 |
| 18 | 1.11 | ± | 0.30 | 0.27 | ± | 0.08 | 0.27 | ± | 0.06 |
| 24 | 1.15 | ± | 0.11 | 0.36 | ± | 0.14 | 0.36 | ± | 0.02 |
| 30 | 1.07 | ± | 0.23 | 0.35 | ± | 0.06 | 0.35 | ± | 0.06 |
| 36 | 1.12 | ± | 0.40 | 0.34 | ± | 0.22 | 0.34 | ± | 0.05 |
| 48 | 1.26 | ± | 0.06 | 0.29 | ± | 0.03 | 0.29 | ± | 0.01 |
| 60 | 1.31 | ± | 0.11 | 0.29 | ± | 0.06 | 0.29 | ± | 0.04 |
| 72 | 1.36 | ± | 0.17 | 0.28 | ± | 0.06 | 0.28 | ± | 0.01 |
| P-value |  |  |  |  |  |  |  |  |  |
| Linear | 0.001 | | | 0.008 | | | 0.840 | | |
| Quadratic | <0.001 | | | 0.0014 | | | 0.178 | | |
| Cubic | <0.001 | | | <0.001 | | | 0.056 | | |
| NFNF, non-fibrous nitrogen fraction; HNF, hemicellulose nitrogen fraction; ADIN, acid detergent insoluble nitrogen; % v.b., Volatile solid basis. | | | | | | | | | |
| ^1^NFNF = total nitrogen – HNF, HNF = neutral detergent insoluble nitrogen – ADIN, and ADIN included nitrogen content of cellulose and lignin content. | | | | | | | | | |
